# ECAP-Controlled Closed-Loop Spinal Cord Stimulation for Chronic Nonsurgical Refractory Back Pain: Subgroup Analysis From Two Prospective Multicenter Clinical Trials

**DOI:** 10.1097/BRS.0000000000005445

**Published:** 2025-07-01

**Authors:** Corey W. Hunter, Jeffrey S. Raskin, Nagy A. Mekhail, Erika A. Petersen, Shivanand P. Lad, Jason E. Pope, Shrif J. Costandi, Leonardo Kapural, Ronald B. Boeding, Ajay Antony, Steven M. Rosen, Robert D. Heros, Dawood Sayed, Sean Li, Ahmed M. Raslan, G Lawson Smith, Johnathan H. Goree, Angela Leitner, Nicole Soliday, Rui V. Duarte, Timothy R. Deer

**Affiliations:** a Ainsworth Institute of Pain Management, New York, NY; b Department of Neurosurgery, Northwestern University Feinberg School of Medicine, Chicago, IL; c Division of Pediatric Neurosurgery, Ann and Robert H. Lurie Children’s Hospital, Chicago, IL; d Department of Pain Management, Cleveland Clinic, Cleveland, OH; e Department of Neurosurgery, University of Arkansas for Medical Sciences, Little Rock, AR; f Department of Neurosurgery, Duke University Medical Center, Durham, NC; g Evolve Restorative Center, Santa Rosa, CA; h Center for Clinical Research, Carolinas Pain Institute, Winston-Salem, NC; i iSpine Clinics, Maple Grove, MN; j The Orthopaedic Institute, Gainesville, FL; k Delaware Valley Pain and Spine Institute, Trevose, PA; l Spinal Diagnostics, Tualatin, OR; m Department of Anesthesiology, University of Kansas School of Medicine, Kansas City, KS; n National Spine and Pain Centers, Shrewsbury, NJ; o Department of Neurological Surgery, Oregon Health and Science University, Portland, OR; p Saluda Medical Pty Ltd, Macquarie Park, New South Wales, Australia; q Department of Health Data Science, University of Liverpool, Liverpool, UK; r The Spine and Nerve Center of the Virginias, Charleston, WV

**Keywords:** closed-loop, evoke-compound action potential, neurophysiological dose metrics, nonsurgical refractory back pain, Persistent Spinal Pain Syndrome Type 1, spinal cord stimulation

## Abstract

**Study Design.:**

Subgroup analysis of patients with chronic nonsurgical refractory back pain (NSRBP) from two prospective multicenter clinical trials to 12-month follow-up.

**Objective.:**

To evaluate pain-related and holistic response, safety events as well as neurophysiological metrics associated with the use of evoked compound action potential (ECAP)-controlled closed-loop spinal cord stimulation (SCS) for patients with chronic back pain without prior surgery.

**Summary of Background Data.:**

Innovations in SCS such as the development of physiological ECAP-controlled closed-loop SCS overcome limitations of traditional, fixed-output SCS for the treatment of NSRBP. The outcomes of closed-loop SCS to 12-month follow-up for patients with NSRBP have not been previously reported.

**Materials and Methods.:**

Patient-reported outcome measures for pain intensity, physical function, health-related quality of life, sleep quality, and emotional function were collected from 68 patients with NSRBP in two prospective multicenter clinical trials. Change in opioid use, its reduction or elimination were assessed at 12-month follow-up. A validated composite outcome measure comprising the different health domains was used to evaluate holistic treatment response through minimal clinically important differences (MCIDs). Objective device metrics provide information on system utilization, loop performance (dose accuracy), and neurophysiological dose metrics.

**Results.:**

At 12 months, 79% of patients reported ≥50% reduction in pain intensity and 48% obtained ≥80% pain relief. Significant improvements in all patient-reported outcome measures assessed were observed at 3 and 12 months. Voluntary reduction or elimination of opioid use was observed in approximately half of the patients that were taking opioids at baseline. System utilization was >80%, dose ratio was >1.3 (*i.e.* 30% above ECAP threshold) with a high-dose accuracy keeping the elicited ECAP within 3.5 μV of the target ECAP set on the system.

**Conclusion.:**

ECAP-controlled closed-loop SCS represents a safe and effective treatment option for patients with NSRBP.

Low back pain is a highly prevalent condition that affected 619 million people globally in 2020.^1^ By 2050 the number of individuals with low back pain is projected to reach 843 million people worldwide, a 36.4% increase from 2020.^1^ Low back pain remains the leading cause of years lived with disability worldwide despite the availability of numerous pharmacological and noninvasive treatment options.^2,3^ Between 32% and 60% of people experience acute pain recurrence or their condition evolves into chronic low back pain.^4,5^

Chronic pain management follows a pathway that begins conservatively and progresses to incrementally more invasive options if the patient does not obtain satisfactory pain/symptom relief.^6^ Despite years of multimodal conventional medical management (CMM) options a proportion of patients do not obtain satisfactory pain relief.^7^ Spinal surgery is typically considered late in the treatment algorithm once all other conservative and minimally invasive options have failed due to the fact that it is invasive, irreversible, and expensive. Furthermore, surgery does not always lead to the anticipated outcome, as approximately one in five patients are expected to experience chronic axial back pain after surgical intervention, or Persistent Spinal Pain Syndrome Type 2 (PSPS-T2), within two years of surgery.^8^ In addition, many of these patients are not indicated for spine surgery or choose not to undergo spine surgery and they have limited treatment options.

Historically, spinal cord stimulation (SCS) relied on patient report of paresthesia overlapping the distribution of pain and was most commonly used for the management of PSPS-T2 with predominant leg pain.^9,10^ The use of SCS for nonsurgical refractory back pain [NSRBP; also known as Persistent Spinal Pain Syndrome Type 1 (PSPS-T1), or chronic back pain without prior surgery/nonsurgical, maiden, or “virgin” back pain], has been less explored due to challenges with the lateral dorsal root entry of the second lumbar fibers (L2), and its deeper position within the thoracic dorsal columns, which hinder adequate coverage of the lower back.^11,12^ Consequently, higher amounts of energy are required at the midline of the spinal cord to activate these fibers, but frequently at the cost of unwanted lower extremity stimulation as well as overstimulation laterally, thus resulting in activation of nearby nerve roots which causes the stimulation to become obtrusive or painful. The stimulation is further confounded by a larger cerebrospinal fluid space at the level of the lower back fibers which results in greater positional variability (*i.e.* too little/much stimulation caused by changes in distance between the leads and the spinal cord as a consequence of position of the patient).^13^

Innovations in neurostimulation paradigms have triggered an interest in the potential of SCS to provide clinically meaningful benefits for patients suffering with NSRBP. One such innovation is the development of physiological-evoked compound action potential (ECAP)-controlled closed-loop SCS; this particular platform enables continuous and automatic, real-time adjustment of the stimulation output of each electrical pulse to adjust the dose to the optimal target to ensure therapeutic accuracy of the therapy.^14–16^ As it pertains to the treatment of back pain, the ability to control the level of dorsal column fiber activation in this way increases the likelihood of these target fibers being activated by each pulse while avoiding the collateral activation of neural tissue that is an unwanted side-effect (such as dorsal root activation) therefore mitigating the challenges described above to more effectively deliver SCS therapy to treat back pain. In addition, with the ability to avoid dorsal root activation, leads may be placed at sites over the dorsal columns that are more lateral, or more caudal, than is typical with conventional open-loop SCS systems.^17^ Such alternative lead positions may overcome the neuroanatomical challenge of dorsal column fibers innervating the back being more lateral when they first enter the dorsal column tracts, and terminating before reaching the level where the leads are able to activate them for therapy provision; stimulating closer to the dorsal column entry point of the target fibers offers the greatest chance of activating them before potential termination. Thus, more lateral and/or caudal lead positions could exploit this anatomic feature to potentially provide improved therapeutic outcomes.

The EVOKE randomized controlled trial (RCT) and ECAP prospective single-arm study recruited patients with chronic, intractable back and leg pain.^18,19^ The EVOKE RCT showed superiority of ECAP-controlled closed-loop SCS over traditional, open-loop SCS in the treatment of chronic, intractable back and leg pain.^16,20,21^ However, findings for closed-loop SCS specific to patients with NSRBP have not been previously reported. This study aims to report the pain-related and holistic response, safety events as well as the neurophysiological metrics that produced the outcomes associated with the use of closed-loop SCS specific to patients with NSRBP.

## MATERIALS AND METHODS

### Study Population

Candidates with chronic, intractable back and leg pain refractory to conservative therapy with a minimum visual analog scale (VAS) score of 60 mm or higher (where 100 mm indicates the worst imaginable pain and 0 mm no pain), who provided written informed consent, were screened for enrollment in the EVOKE and ECAP studies. The full eligibility criteria are presented in the respective protocols.^18,19^ The EVOKE study was a multicenter, participant, investigator, and outcome assessor-blinded, parallel-arm RCT conducted at 13 investigation sites throughout the United States under an Investigational Device Exemption to gain US Food and Drug Administration (FDA) approval (registered on Clinicaltrials.gov, October 5, 2016; NCT02924129). The ECAP study was a prospective, multicenter, single-arm study conducted at 25 sites in the United States to collect routine clinical care data (registered on Clinicaltrials.gov, March 24, 2020; NCT04319887). The studies were conducted in compliance with ethical and regulatory requirements and were approved by local ethics committees before subject enrollment.

The current analysis considers the subgroup of patients with NSRBP recruited to the EVOKE RCT (only subjects that received closed-loop SCS) and ECAP study. Diagnosis involved information about patient medical history, physical examination, and consideration of previous treatments attempted, with NSRBP participants defined as participants with refractory low back pain, with or without leg pain, who were spine surgery naïve.

### Trial and Implant Procedure

All patients underwent a temporary SCS trial in accordance with best practices.^22^ Patients with 50% or more VAS score reduction in overall back and leg pain were eligible for permanent implantation in the EVOKE study^18^ and according to standard of care procedures in the ECAP study.^19^ During the temporary trial and permanent implant procedures, two percutaneous leads were implanted in the dorsal epidural space as per standard practice. Most patients had the tip of the lead placed between T6 and T9. The neuromodulation system (Evoke SCS System; Saluda Medical Pty Ltd, Macquarie Park, Australia) provided physiological, ECAP-controlled closed-loop SCS and the ability to measure the neural activation elicited by each stimulation pulse. Physiological closed-loop control system (PCLCS) is a proportional-integral-derivative controller, which minimizes the difference between the measured ECAP amplitude and the ECAP amplitude target by automatically varying the stimulation current amplitude in real time in a frequency dependent manner to maintain consistent therapy. The SCS system used in the current study meets the FDA and American Society of Pain and Neuroscience definition of a PCLCS.^23–25^

### Outcomes

The EVOKE and ECAP studies assessed outcome measures at baseline (preimplantation), 3 and 12 months postimplantation. EVOKE RCT results for the full cohort through 36 months are presented elsewhere.^16,20,21,26^

### Patient-Reported Outcome Measures

Pain relief was assessed by determining the percent change from baseline in a 100 mm visual analog scale (VAS) ranging from 0 (no pain) to 100 (worst possible pain),^27^ and the proportion of patients with ≥50% and ≥80% reduction in pain intensity. Physical function was measured using the Oswestry Disability Index (ODI).^28^ Health-related quality of life (HRQoL) was assessed using the EQ-5D-5L in EVOKE and PROMIS-10 Global Health in ECAP, with responses converted into single (utility) indices using the US value set crosswalks to EQ-5D-3L.^29,30^ Sleep quality was evaluated using the Pittsburgh Sleep Quality Index (PSQI) in EVOKE and PROMIS-29 in ECAP, with responses mapped to the PSQI.^31^ Emotional function was estimated using the Profile of Mood States-Brief (POMS-B) tool.^32^ Clinically meaningful differences [literature defined minimal clinically important differences (MCIDs)] from normative values were used to characterize baseline dysfunction (*i.e.* cumulative MCID from the norm). Change in opioid use, its reduction or elimination were assessed at 12-month follow-up.

### Holistic Treatment Response

Treatment response was assessed by attaining MCIDs for VAS, ODI, POMS, PSQI, and EQ-5D-5L. The breadth of treatment response refers to the number of domains in which at least one MCID was achieved while the depth of treatment response refers to the number of MCIDs obtained in each domain. Holistic treatment response was determined for each patient based on attaining at least one MCID improvement in all domains that were impaired at baseline when compared with normative US values.^33,34^ In addition, the total amount of MCIDs achieved were calculated for each domain and pooled for all domains to derive a cumulative responder score. The Holistic MCID, a validated composite outcome measure, considered the cumulative responder score adjusted for the number of impaired domains at baseline for each patient.^35^

### Objective Device Metrics

Objective device metrics collected provide information on system utilization, loop performance (dose accuracy), and various neurophysiological dose metrics.

#### System Utilization

System utilization was defined as the proportion of time the system was on for the week before the scheduled visit.

#### Neurophysiological Dose Metrics

Percent time above ECAP threshold was defined as the proportion of time a patient received stimulation above their individual ECAP threshold, out of the total stimulation time.

ECAP dose, used interchangeably with neural dose, is defined by the median ECAP level using the normalized ECAP amplitude (μV).

The dose ratio is determined by the estimated current (mA) at the median ECAP level divided by the current (mA) at the ECAP threshold.^36^ The dose ratio allows for both the individualization of a patient’s neural dose and comparison across patients, using their ECAP threshold and their spinal cord sensitivity, as defined by the slope of the ECAP amplitude to current curve. This metric normalizes for distance between the electrode and the spinal cord and the distances between the stimulation and recording electrodes. The dose ratio reflects the amount of dorsal column activation elicited by stimulation with respect to threshold levels. For example, if the ECAP dose generated is right at ECAP threshold, this value will be 1. The ECAP dose generated at a stimulation level 40% higher than ECAP threshold will have a dose ratio value of 1.4.

#### Neurophysiological Dose Accuracy

The loop performance is characterized by the ability of the system to minimize the error between the ECAP target amplitude established in the clinic and the measured ECAP amplitude. The dose accuracy is defined by the root mean square error (RMSE) of recorded ECAPs compared with the ECAP target and is based on µV of deviation from the ECAP target.

##### Adverse Events

All adverse events (AEs) were reported by the investigators throughout the study and reviewed and adjudicated by an independent clinical adjudication committee.

### Statistical Analysis

Descriptive statistics are provided as mean (SD), median (IQR), or number of observations (percentage), as appropriate. Differences between treatment groups were evaluated using two-sample *t* test for continuous variables and the Fisher exact test for categorical variables. Changes from baseline were evaluated using paired-sample *t* test for continuous variables. Statistical significance was judged at the 5% level. Statistical analyses were conducted using SAS statistical software V.9.4 (SAS Institute).

## RESULTS

**Figure 1:**
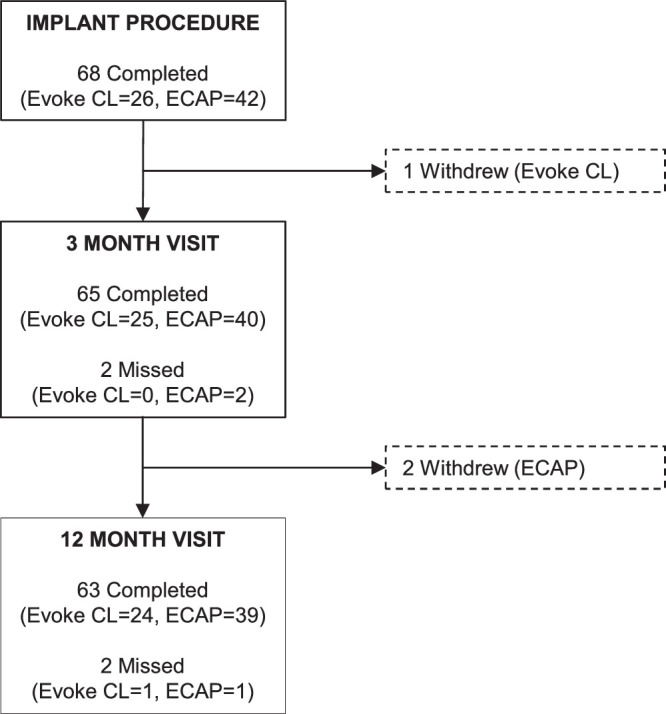
Study participation flow diagram.

Sixty-eight patients with NSRBP received ECAP-controlled closed-loop SCS following a temporary trial phase, with 65 and 63 patients included through 3 and 12 months, respectively (Fig. 1). Baseline demographics and other patient characteristics for EVOKE and ECAP studies were generally well balanced (Table 1). Patients included in the ECAP study were older than those in the EVOKE study. Patients mean age was 59±12 years, with similar representation by sex [female n=37 (54.4%)], a mean duration of pain of 16±13 years and body mass index of 32.4±6.1 kg/m^2^.

**TABLE 1:** Baseline Demographics and Characteristics for CL-SCS Patients

	EVOKE (N=26)	ECAP (N=42)	Total (N=68)
Age (yr)	52.8±8.2	62.8±12.5	59.0±12.0
Sex			
Male	14 (53.8)	17 (40.5)	31 (45.6)
Female	12 (46.2)	25 (59.5)	37 (54.4)
BMI (kg/m^2^)	32.6±5.7	32.2±6.4	32.4±6.1
Duration of pain (yr)	17.4±11.9	15.0±13.4	15.9±12.8
Baseline pain medication usage			
Opioids	18 (69.2)	20 (47.6)	38 (55.9)
Nonopioids*	20 (76.9)	36 (85.7)	56 (82.4)
Most commonly reported pain etiologies (not mutually exclusive)			
Radiculopathy	26 (100)	36 (85.7)	62 (91.2)
Degenerative disc disease	15 (57.7)	28 (66.7)	43 (63.2)
Spondylosis	19 (73.1)	28 (66.7)	47 (69.1)
Spinal Stenosis	10 (38.5)	23 (54.8)	33 (48.5)

Data are mean±SD or n (%).

Most commonly reported pain etiologies=those present in >40% of study population

* Nonopioid pain medication classes include: anticonvulsant, antidepressant, local anesthetic, muscle relaxant, NSAIDs, and other pain medications.

### Baseline Dysfunction

**Figure 2:**
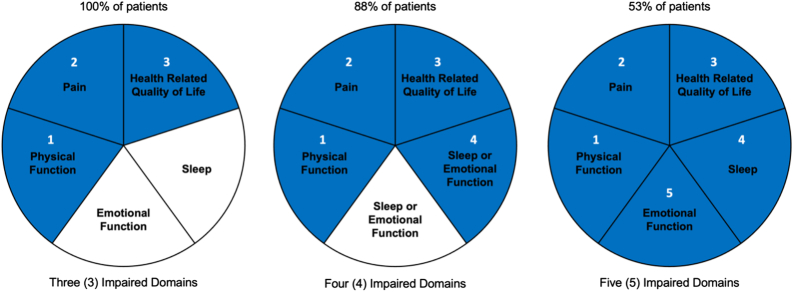
Proportion of patients in the combined EVOKE and ECAP studies population reporting impairments in the different domains at baseline. Hundred percent of patients presented scores worse than normative population values at baseline for pain intensity, physical function, and health-related quality of life. Eighty-eight percent of patients presented scores worse than normative population values for four domains (pain intensity, physical function, health-related quality of life, and sleep or emotional function), and 53% for all five domains.

**Figure 3:**
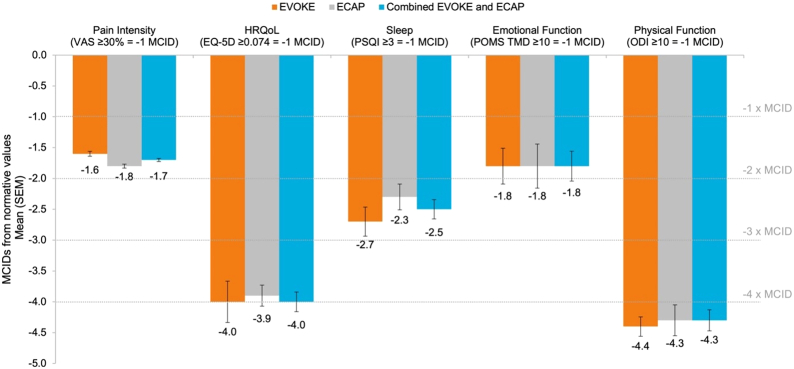
Baseline impairment characterized by MCIDs from normative population values for the different outcome domains. Published normative values considered were: baseline pain intensity (VAS) <40 mm,^37,38^ health-related quality of life (EQ-5D) utility score ≥0.830,^39^ sleep (PSQI) ≤6.3,^40^ emotional function (POMS) ≤17.7,^32^ physical function (ODI) ≤10.19.^28^

All included patients presented scores worse than normative population values at baseline for pain intensity (VAS), physical function (ODI), and HRQoL (EQ-5D-5L); 89% were also impaired for sleep quality (PSQI) and 69% for emotional function (POMS). Eighty-eight percent of patients presented scores worse than normative population values for four domains and 53% for all five domains (Fig. 2). Mean MCIDs from normative population values ranged from 1.7±0.2 for pain intensity to 4.3±1.4 for physical function (Fig. 3). Overall dysfunction represented by cumulative MCIDs from the norm at baseline was 13.1±4.0.

### Patient-Reported Outcome Measures

**Figure 4:**
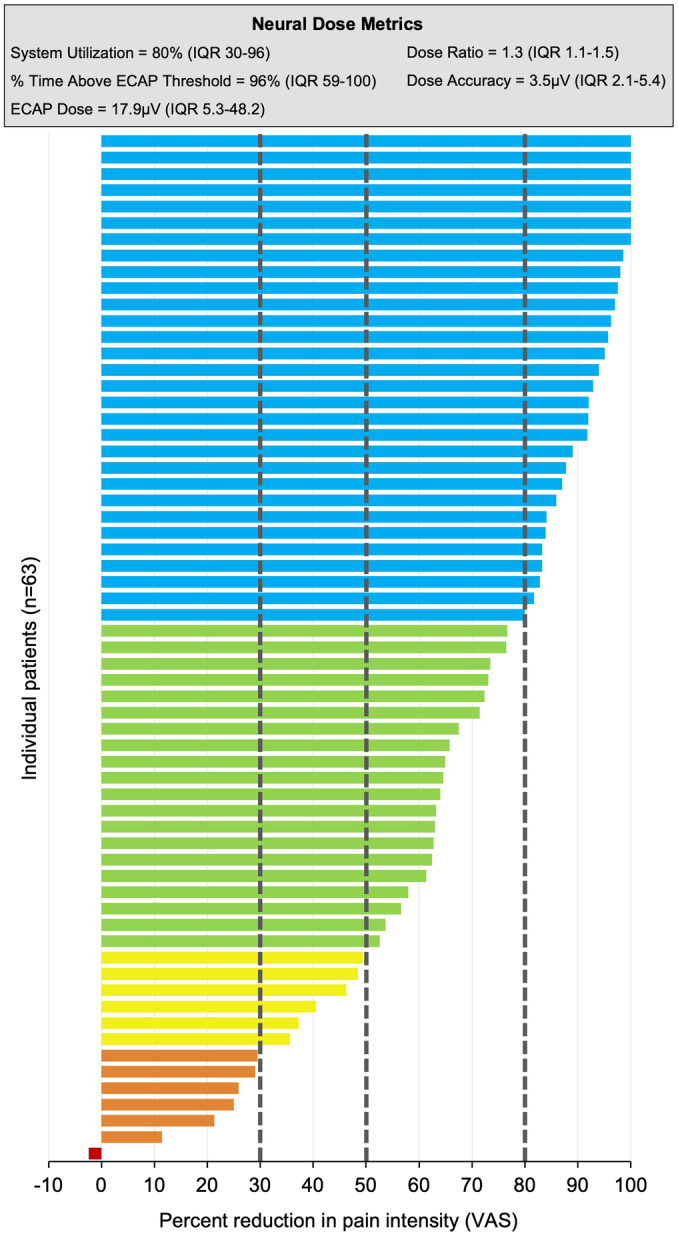
Tornado diagram with individual patient percent change from baseline to 12 months and neural dose metrics that produced these outcomes. Percentage of patients with <30% improvement=11.1%; ≥30% improvement=88.9% with at least one MCID for pain intensity; ≥50% improvement=79.4% responders; ≥80% improvement=47.6% high responders.

Pain intensity significantly decreased from a baseline VAS of 84.9±10.7 to 23.5±23.4 at 3 months and 24.9±22.2 at 12 months (both *P*<0.001). At 12 months, 79% of patients reported ≥50% reduction in pain intensity and 48% obtained ≥80% pain relief (Fig. 4). Significant improvements in all other patient-reported outcome measures assessed were observed at 3 and 12 months for ODI, POMS, EQ-5D-5L, and PSQI (Table 2). At 12 months, 83% of patients considered their health status to be “very much improved” or “much improved” following closed-loop SCS, with 91% of patients “very satisfied” or “satisfied” with pain relief and therapy and 89% of patients reporting they would “recommend” or “strongly recommend” the therapy.

**TABLE 2:** Patient-Reported Outcome Measures

Patient-reported outcomes (PRO)	Baseline (n=68)	3 months (n=65)	12 months (n=63)
Visual analog scale (VAS) pain			
VAS pain intensity	84.9±10.7	23.5±23.4	24.9±22.2
Change from baseline	—	61.6±22.6*	60.0±22.5*
Percent change from baseline	—	73.1±25.7^*^	71.0±25.6*
≥50% reduction	—	53/64 (82.8)	50/63 (79.4)
≥80% reduction	—	29/64 (45.3)	30/63 (47.6)
Oswestry Disability Index (ODI)			
ODI score	52.0±10.2	28.1±15.9	31.5±16.1
Change from baseline	—	23.9±16.7*	20.6±15.9*
Percent change from baseline	—	44.4±32.0*	38.7±31.5*
Profile of mood states (POMS) total mood disturbance (TMD)			
POMS TMD score	22.4±19.2	6.9±17.1	10.6±16.6
Change from baseline	—	14.9±15.6*	11.1±16.5*
EQ-5D-5L index			
EQ-5D-5L index score	0.537±0.099	0.699±0.137	0.683±0.130
Change from baseline	—	0.162±0.152*	0.145±0.155*
Pittsburgh Sleep Quality Index (PSQI)			
PSQI score	12.5±4.8	8.8±3.8	9.1±3.8
Change from baseline	—	3.7±4.8*	3.3±5.0*
Patient global impression of change (PGIC)			
Very much improved or much improved	—	55/64 (85.9)	52/63 (82.5)
Patient satisfaction			
Very satisfied or satisfied with pain relief	—	56/64 (87.5)	57/63 (90.5)
Very satisfied or satisfied with therapy	—	57/64 (89.1)	57/63 (90.5)
Strongly recommend or recommend therapy	—	59/64 (92.2)	56/63 (88.9)
Opioid reduction or elimination if taking at baseline			
Percent change from baseline	—	—†	31.7 (39.8)*
Opioid reduction/elimination	—	—†	16/34 (47.1)

Data are mean±SD or n/N (%).

* *P*<0.001

† Participants in the EVOKE study were asked not to change medications before three-month follow-up

### Holistic Treatment Response

The mean improvement in each domain was greater than the clinically meaningful threshold (*i.e.* 1 MCID) at 3 and 12 months (Table 3). For VAS, ODI, and EQ-5D-5L, >2 MCIDs were reported at 3 and 12 months. The cumulative responder score which reflects the total number of MCIDs obtained across all domains was 9.7±5.8 and 8.7±5.9 MCIDs at 3 and 12 months, respectively. The Holistic MCID which adjusts the cumulative responder score by the number of impaired baseline domains was 2.1±1.2 and 1.8±1.2 at 3 and 12 months, respectively. A large proportion of patients reported clinically meaningful improvements in ≥1, ≥2, and ≥3 domains; >50% of patients obtained MCIDs for at least four domains reported as being worse than normative population values at baseline (Table 4).

**TABLE 3:** Proportion of Responders and Cumulative MCIDs at 3 and 12 months

Outcomes	3-month	12-month
Pain intensity responders (VAS ≥30%)	59/64 (92.2%)	56/63 (88.9%)
Pain intensity mean (SD) number of MCIDs	2.4 (0.9)	2.4 (0.9)
Physical function responders (ODI score ≥10)	52/64 (81.3%)	45/62 (72.6%)
Physical function mean (SD) number of MCIDs	2.6 (1.9)	2.2 (1.9)
QoL responders (EQ-5D-5L Index Score ≥0.074)	49/65 (75.4%)	39/63 (61.9%)
QoL mean (SD) number of MCIDs	2.2 (2.1)	2.0 (2.1)
Sleep responders (PSQI ≥3)	40/63 (63.5%)	37/61 (60.7%)
Sleep mean (SD) number of MCIDs	1.3 (1.5)	1.2 (1.6)
Emotional function responders (POMS TMD Score ≥10)	31/44 (70.5%)	29/43 (67.4%)
Emotional function mean (SD) number of MCIDs	1.9 (1.7)	1.6 (1.5)
Cumulative responder score, mean (SD)	9.7 (5.8)	8.7 (5.9)
Holistic MCID	2.1 (1.2)	1.8 (1.2)

MCID indicates minimal clinical important difference; ODI, Oswestry Disability Index; POMS, Profile of Mood States; PSQI, Pittsburgh Sleep Quality Index; QoL, quality of life; SCS, spinal cord stimulation; TMD, total mood disorder; VAS, visual analog scale.

**TABLE 4:** Multimodal Treatment Response in NSRBP Patients With Closed-Loop SCS

	3-month, n/N (%)	12-month, n/N (%)
Responders in ≥1 impaired domain	64/65 (98.5)	59/63 (93.7)
Responders in ≥2 impaired domains	57/65 (87.7)	51/63 (81.0)
Responders in ≥3 impaired domains	53/65 (81.5)	45/63 (71.4)
Responders in ≥4 impaired domains	38/64 (59.4)	33/62 (53.2)
Responders in 5 impaired domains	19/41 (46.3)	18/41 (43.9)

MCID indicates minimal clinical important difference; ODI, Oswestry Disability Index; POMS, Profile of Mood States; PSQI, Pittsburgh Sleep Quality Index; SCS, spinal cord stimulation; VAS, visual analog scale.

There was a decrease in the proportion of patients with scores worse than normative population values at 12-month follow-up compared with baseline. At baseline 100% of patients presented scores worse than normative population values for three domains, this proportion was 76% at 12 months; for four domains, the decrease was from 88% at baseline to 29% at 12 months; and for five domains, the decrease was from 53% at baseline to 8% at 12 months.

### Objective Device Metrics

The neurophysiological metrics that produced the reported outcomes are presented in Table 5. The performance of the feedback loop resulted in high-dose accuracy by keeping the elicited ECAP within 3.5 μV of the target ECAP set on the system at 3 and 12 months. The SCS system utilization was >80%, percent time stimulating above ECAP threshold was >95%, ECAP dose was ≥17.9 μV, and the dose ratio was >1.3 (*i.e.* 30% above ECAP threshold) at 3 and 12 months follow-up.

**TABLE 5:** Neural Activation in NSRBP Patients With Closed-Loop SCS

Device metric	3-month	12-month
Frequency of use		
System utilization (% time on)	88.2 (69.4–96.6)	80.4 (30.3–96.2)
Neurophysiological dose metrics		
Percent time above ECAP threshold (out of total stimulating time)	97.1 (73.2–100.0)	95.8 (59.0–99.9)
ECAP dose (normalized median ECAP amplitude, μv)	22.9 (7.1–53.8)	17.9 (5.3–48.2)
Dose ratio (estimated current at median ECAP/ECAP threshold current)	1.32 (1.06–1.47)	1.32 (1.10–1.47)
Loop performance		
Dose accuracy [in-clinic deviation from ECAP target, RMSE (μv)]	3.1 (2.3–4.8)	3.5 (2.1–5.4)

Median (interquartile range).

### Adverse Events

Over the course of 12 months, 10 study-related AEs were observed in 8/68 (11.8%) patients (Supplementary Material 1, Supplemental Digital Content 1, http://links.lww.com/BRS/C761). The most common AE was lead migration [two events in two (2.9%) patients]. There was one study-related serious AEs event (wound infection) in one (1.5%) patient. There were no device explants due to loss of efficacy.

## DISCUSSION

The results of this study demonstrate that ECAP-controlled closed-loop SCS is a safe and effective treatment option for patients with NSRBP. The treatment limitations of CMM and the lack of spine surgery indications makes SCS for NSRBP intriguing. We present for the first time the neurophysiological metrics associated with the clinical outcomes produced by ECAP-controlled closed-loop SCS in this patient population. Over the 12-month period, patients received supra-ECAP threshold therapy >95% of the time, at a dose ratio level of 1.32 (*i.e.* 32% above ECAP threshold), and system utilization of >80%. The feedback loop was optimized and performing within 3.5 μV of the ECAP target. In this large cohort of NSRBP patients that received closed-loop therapy, we observed statistically and clinically significant improvements in pain intensity, physical function, HRQoL, sleep quality, emotional function, and opioid use at 12 months follow-up. The Holistic MCID score at 12 months indicates that on average, patients obtained approximately two MCIDs in domains that were worse than normative values at baseline. In addition, 82.5% of patients considered their health status to be “much improved” or “very much improved” and 90% of patients were satisfied or very satisfied with the therapy. Of critical importance, voluntary reduction or elimination of opioids in half the patients provides further demonstration that ECAP-controlled closed-loop SCS not only provides significant pain relief but is an effective alternative to long-term opioid dependence in this challenging patient population.

The outcomes observed in this study support the recent network meta-analysis demonstrating superiority of closed-loop SCS compared with fixed-output SCS and CMM for an NSRBP population at six months follow-up.^41^ In the network meta-analysis, fixed-output consisted of burst, high-frequency, and ECAP-guided open-loop SCS. Recent RCT evidence reported similar findings for improvements in pain intensity, physical function, HRQoL, impression of change, satisfaction, and opioid reduction across multiple waveforms.^42–46^ A recent RCT observed superior outcomes for differential target multiplexed SCS compared with conventional SCS.^46^ The results support relative limitations for conventional SCS for patients with NSRBP. Both differential target multiplexed SCS and conventional SCS are types of fixed-output SCS; as such, it would not be feasible to use this evidence to update the recent network meta-analysis of SCS for NSRBP.

A well-known limitation of SCS evidence is the lack of information on device metrics from the majority of device manufacturers.^33,47^ As previously stated, the omission of these data precludes any potential reproducibility of SCS studies and outcomes for patients.^26,47,48^ The neurophysiological metrics in the current study were within the ranges recently found to provide maximal analgesic effect in a population with chronic, intractable back and leg pain.^49^ It is plausible that optimization of neurophysiological metrics would generate incremental improvements in all holistic domains. Research is ongoing to evaluate the potential for a dosing regimen to optimize clinical benefit (NCT06229470).

With the use of a validated composite outcome for assessment of the chronic pain experience,^35^ we observed a holistic treatment response and >1 MCID in pain intensity, physical function, HRQoL, sleep quality, emotional function at 12 months. A Cochrane review concluded that there was probably no benefit of SCS over placebo on pain, function, or HRQoL in the medium term for people with low back pain.^50^ The limitations and errors in that review have been previously described.^51–53^ Of note, five of eight studies included in the Cochrane review evaluated experimental types of SCS that are not used in current clinical practice and the patient population does not reflect patients with NSRBP. Despite the repercussions that followed the publication, we consider that the Cochrane review has limited-to-no value for the purposes of decision-making.

Numerous economic evaluations have shown that SCS is a cost-effective treatment option, particularly for the management of PSPS-T2 and complex regional pain syndrome.^54–56^ Health economic evidence on the use of SCS for NSRBP has only recently started to emerge. Recent evidence from before-and-after retrospective studies observed reductions in health care use with high-frequency SCS when compared with CMM (before implant),^57,58^ and results from a trial-based economic evaluation suggested that high-frequency SCS was dominant compared with CMM, although the costs of the implant and of the trial procedure were not included in the analysis.^59^ These findings, however, are consistent with those reported in another study which found that traditional, fixed-output SCS was dominant compared with CMM.^41^ Further, this study also reported that closed-loop SCS was cost-saving and generated more quality-adjusted life years (*i.e.* dominant treatment strategy) when compared with fixed-output SCS and CMM.^41^

It is important to note that the authors do not suggest that SCS should be provided to all patients with low back pain that have not had surgery. The population included in the current study consisted of patients with chronic back and leg pain that had not resolved with conservative treatment options over more than a decade on average. The time required to evaluate treatment response to conservative options should be minimized to prevent undue suffering and to minimize the long-term use of opioids. There are ongoing efforts to characterize the population with NSRBP that should be considered for SCS.^60^ Ineligibility for spinal surgery is unlikely to be a criterion for appropriateness for SCS. Irrespective of suitability or not for spinal surgery, patients may prefer to defer surgery in favor of an intervention that is reversible, like SCS. In the United Kingdom, ∼5000 new patients annually experience pain despite spinal surgery, with each new annual cohort estimated to cost the health care system more than £70 million over the first 10 years alone.^8^ In the United States, although only a small proportion of patients with new onset low back pain with or without lower extremity pain are estimated to receive spinal surgery, these account for 29.3% of the total 12-month costs of care for low back pain following diagnosis ($784 million) suggesting that spine surgery is a significant driver of health care expenditure.^61^ Given the mounting evidence on novel types of SCS for NSRBP, the irreversibility, cost and incidence of PSPS-T2, we posit that the position of SCS in the treatment pathway should be reassessed, potentially ahead or at least in line with spinal surgery.

### Strengths and Limitations

We report a comprehensive, holistic assessment of closed-loop SCS treatment response for patients with NSRBP and the neurophysiological metrics that produced the outcomes observed. The current study is limited by the absence of a comparator group; however, a previous study demonstrated that closed-loop SCS resulted in statistically and clinically significant improvements for patients with NSRBP when compared with fixed-output SCS and CMM.^41^ Although the current study is a post hoc analysis of a subgroup of patients from the EVOKE and ECAP studies, the patient demographics and characteristics were generally similar to other reports of SCS for NSRBP and recently published guidelines of neurostimulation for this patient population.^42–46,62^ Individual patient data meta-analysis may help identify subgroups within the broader NSRBP population that may obtain more benefits from SCS. Neural biomarkers of spinal cord activation can assist in the identification of those patients more likely to obtain better outcomes.^48,49^ In addition, mapping of different types of NSRBP to ICD-10-CM and ICD-11 codes has been performed to assist with reimbursement.^60^

## CONCLUSION

Physiological ECAP-controlled closed-loop SCS represents a safe and effective treatment option for patients with NSRBP. In addition to pain-related outcomes, the comprehensive holistic assessment and neurophysiological metrics add to the evidence-base in support of SCS for this patient population.

Key Points

- ECAP-controlled closed-loop SCS produced clinically meaningful improvements in multiple health-related domains at 12-month follow-up for patients with nonsurgical refractory back pain (NSRBP).
- The neurophysiological metrics observed were within the ranges recently found to provide maximal analgesic effect in a broader chronic pain population.
- The results of this study add to the evidence supporting the use of spinal cord stimulation for patients with NSRBP.

## Supplementary Material
