# Supplementary material for: ECAP-Controlled Closed-Loop Spinal Cord Stimulation for Chronic Nonsurgical Refractory Back Pain: Subgroup Analysis From Two Prospective Multicenter Clinical Trials
Source: Spine (Phila Pa 1976). 2025 Jul 1;50(23):1637–47. doi: 10.1097/BRS.0000000000005445 (PMC12594148; doi:10.1097/BRS.0000000000005445)
Supplement: Supplementary file 1 [file brs-50-1637-s001.docx]

**Supplementary material 1**

**Study-related adverse events through 12 months**

|  | **Events**  **n** | **Subjects**  **N=68**  **n (%)** |
| --- | --- | --- |
| **Total Adverse Events** | **10** | **8 (11.8%)** |
| Lead Migration | 2 | 2 (2.9%) |
| Dural Puncture | 1 | 1 (1.5%) |
| Inadequate Lead Placement | 1 | 1 (1.5%) |
| Low Back Pain | 1 | 1 (1.5%) |
| Muscle Spasm or Muscle Cramp | 1 | 1 (1.5%) |
| Nausea and/or vomiting | 1 | 1 (1.5%) |
| Seroma or Drainage | 1 | 1 (1.5%) |
| Wound Dehiscence | 1 | 1 (1.5%) |
| Wound Infection | 1 | 1 (1.5%) |
